# Effects of Lignin Fractionation on the Mechanical and Thermo-Oxidation Properties of SSBR/Silica Composites

**DOI:** 10.1021/acssusresmgt.5c00496

**Published:** 2025-11-21

**Authors:** Onur Nuri Arslan, Xiao Hu, Yanxi Shi, Haifeng Liu, Wai Hin Lee, Ming Li, Chaoying Wan

**Affiliations:** † International Institute for Nanocomposites Manufacturing (IINM), WMG, 2707 University of Warwick, Warwick CV4 7AL, U.K.; ‡ 91599 Nanjing Tech University, Nanjing, Jiangsu 211816, China

**Keywords:** lignin fractionation, rubber, thermo-oxidation, nonlinear viscoelasticity

## Abstract

Lignin is a polyphenolic compound extracted from plant pulps in the paper industry. It has attracted significant interest for the rubber industry due to its inherent antioxidant function and reinforcement effect. However, the structural heterogeneity and poor solubility of lignin have limited its applications. This study systematically investigated the chemical composition, molecular weight, and radical scavenging capability of four commercial lignin products and their fractionated portions, derived through sequential fractionation processes. The first fraction (F1) of hardwood lignin (UPM) has the lowest molecular weight (*M*_w_ = 2700) and yield (6%) but the highest radical scavenging activity (RSA = 38.45%). In contrast, F1 of enzymatic hydrolysis lignin (EHL) obtained the highest yield (44%) with a moderate RSA (27.60%), slightly exceeding that of raw EHL (EHL F0). EHL F0 and F1 were further compounded with SSBR/silica for evaluating their effects on the rubber composites through mechanical, rheological, and thermo-oxidant characterization. The addition of 2∼10 phr EHL F0 or F1 shows a semi-reinforcement effect without deteriorating the nonlinear dynamic behavior of the rubber composites. The small loadings of EHL F0 or F1 showed faster oxidation induction than the commercial antioxidant *N*-(1,3-dimethylbutyl)-*N*′-phenyl-*p*-phenylenediamine (6PPD) in an oxygen environment but better long-term thermo-oxidant resistance in hot air. The dispersion and compatibility of lignin with SSBR play decisive roles in performance enhancement.

## Graphical abstract

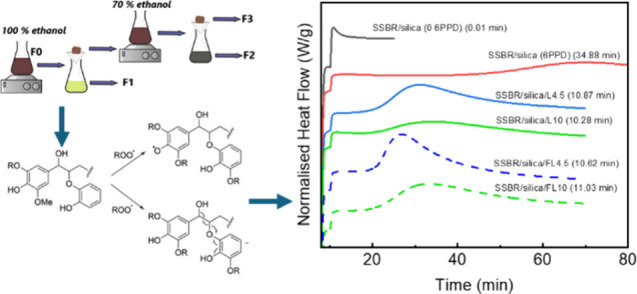

## 1 Introduction

Rubber aging and degradation remain a persistent challenge in the rubber industry, primarily due to oxidative degradation. When exposed to heat or UV radiation, reactive sites, such as the unsaturated double bonds, undergo radical cleavage and chain scission, leading to cracking and mechanical deterioration. Synthetic antioxidants, such as phenolic derivatives and aromatic amines, are commonly used in commercial rubbers; however, the toxicity of these antioxidants, such as *N*-(1,3-dimethylbutyl)-*N*′-phenyl-*p*-phenylenediamine (6PPD) and the oxidized byproduct, 6PPD-quinone (6PPDQ), has been identified as a severe ecological hazard, threatening human and environmental health.

In this context, lignin has been considered as a promising bio-based alternative. Its substituted phenolic moieties offer inherent radical scavenging capacity. Recent studies revisited the mechanisms for antioxidation of lignin and revealed its structure–property relationship, in contrast to the ambiguous ingredients in the past.^1,2^ Lignin constitutes 15∼30 % of wood-derived biomass and is traditionally obtained as a byproduct in pulp and paper manufacturing. Hence, lignin has varied chemical and physical properties depending on its origin (softwood, hardwood, or grass) and isolation process (kraft, sulfite, or organosolv methods). Lignin consists of three primary phenylpropanoid units, *p*-hydroxyphenyl (H), guaiacyl (G), and syringyl (S), which are interconnected through ether (β-O-4, α-O-4) and carbon–carbon to form polydisperse phenolic molecules. The relative content of these units also results in variation in chemical reactivity, such as softwood lignin is predominantly G-type, and hardwood lignin contains both G and S units. To address the inherent polydispersity of lignin, sequential solvent extraction has been applied to refine the chemical structures, known as fractionation. This technique separates and fractionates lignin utilizing different solubilities in organic solvent/water mixtures (e.g., acetone/water or ethanol/water gradients) to regulate molecular weight distribution and chemical functionality as well as improve homogeneity of phenolic content in the products. Each fraction exhibits distinct physical and chemical properties, enabling targeted applications in polymer composites, adhesives, or as antioxidant additives in the desired systems.^3,4^

In the rubber industry, lignin was originally used as a filler alternative to silica or carbon black, but it exhibited less reinforcing effect than silica or carbon black even at high contents.^5^ Yu et al. investigated the influence of partial replacement of silica filler with lignin for natural rubber (NR) compounding and found that the combination of lignin/silica did not affect the mechanical properties but reduced the Payne effect and improved processability, antiaging, and antiflex cracking of the composites. An optimal filler combination of 20 phr lignin and 30 phr of silica was reported to produce balanced mechanical properties of the NR composites.^6^ Qiu et al. prepared the lignin/silica hybrid filler via in situ assembly of diethylamine-grafted lignin and silica and demonstrated that the reinforcement of the hybrid filler was highly dependent on the filler morphology and the interfacial interactions with the rubber matrix.^7^ In 2018, Hait and Das et al. prepared polybutadiene rubber (BR)/lignin composites through a selection of rubber curing ingredients via a high-temperature multi-step melt-mixing process above the glass transition temperature of lignin.^8^ The mechanical properties of the BR/lignin composites containing 50 phr kraft lignin were superior to BR/carbon black composites or BR/silica–silane systems, demonstrating a promising reinforcement potential of lignin.^8^ In 2024, they utilized a thermo-chemomechanical approach to incorporate lignin nanoparticles into SSBR/BR, and 3-aminopropyl triethoxysilane (APTES) was specifically added into the compounds under high temperatures during the compounding process. The resulting vulcanizates showed mechanical and fracture performance comparable to a conventional silica–silane-based tire formulation, with improved crack growth resistance under low tearing energy conditions.^9^ Liu et al. studied the antioxidant properties of lignin within a natural rubber matrix. They found that using graphene oxide as a carrier significantly improved the dispersion of alkali lignin, thereby enhancing its antioxidant efficiency of natural rubber composites, demonstrating a 14.5% increase in tensile strength retention and a 15.9% improvement in elongation at break after thermal aging.^10^ Despite the potential of lignin for green filler applications, the main challenge lies in its poor interfacial compatibility with polymers, melt-flow properties, and ambiguous chemical structures.

The stabilization of rubber by lignin was first reported by Murray and Watson using lignin derived from unoxidized soda pulp in 1948.^11^ Zhao et al. utilized gallic acid, sinapic acid, and α-tocopherol as representative lignin fragment models and studied their radical scavenging ability through a combined modeling and experimental method.^12^ They found that these compounds had lower O–H bond dissociation energies compared to 6PPD, indicating a greater potential to donate hydrogen atoms and neutralize free radicals. After being aged in ozone at 40 °C for 72 h, the α-tocopherol-containing compound exhibited the best performance, retaining 70% of elongation and 35% tensile strength, which was attributed to its lower mobility and better compatibility with the SSBR matrix. Sinapic acid performed similarly to 6PPD, while gallic acid showed the worst performance due to its lower affinity with the polymer matrix. The antioxidation mechanism of lignin is still in debate, owing to the ill-defined chemical structures of lignin. It is generally considered that the phenolic moiety donates a H radical to form a stable oxygen-centered radical, which further rearranges to a quinone-like moiety.^13^ In addition, radical species can also be captured via β-hydrogen abstraction and subsequent chain scission to form inactive species, as shown in Scheme 1.

**Scheme 1:**
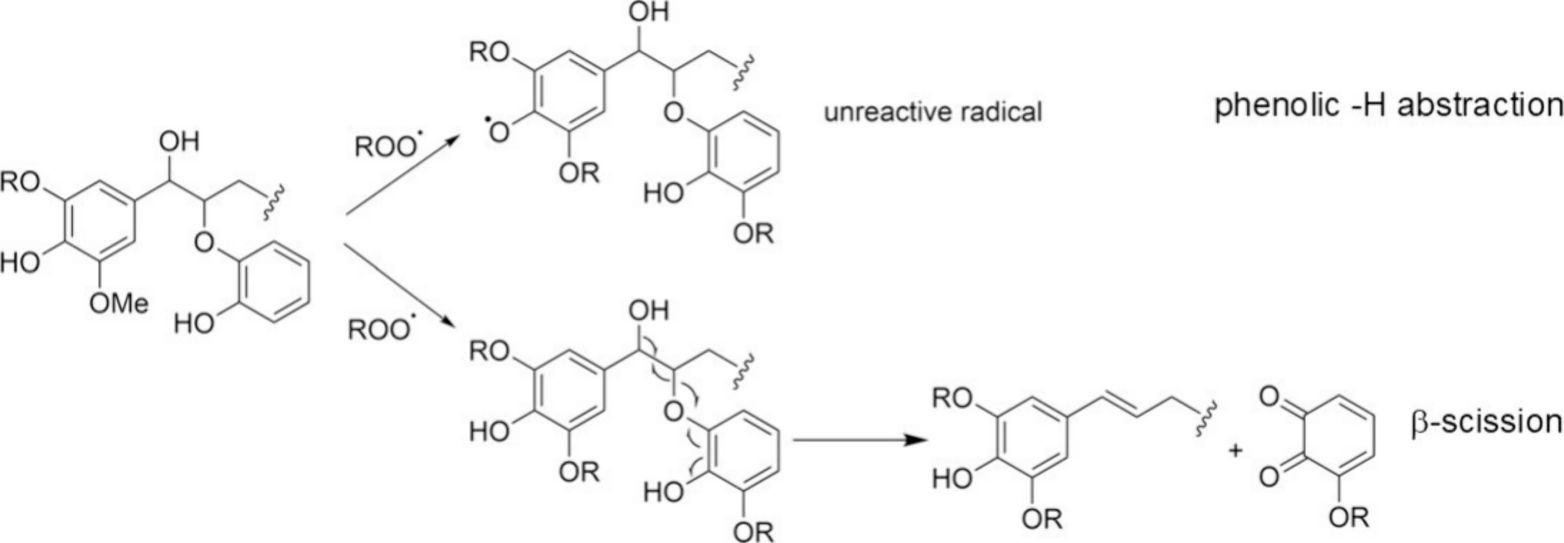
Proposed mechanism of radical scavenging by lignin fragments

Integrated with lignin fractionation into rubber, Xiao et al. compared ethanol- and ethyl acetate-fractionated alkali lignin in styrene–butadiene rubber and found that the oxidation induction time (OIT) was significantly prolonged from 5.4 to 53 min for ethanol-extracted lignin and over 60 min for ethyl acetate-extracted lignin, which was comparable to the industrial benchmark Irganox 1010 (tetrakis-BHT derivatives).^14^ Cheng et al. demonstrated that fractionation enhances the antioxidant activity of lignin due to the reduced molecular weight and, more significantly, the removal of carbohydrate impurities and the consequent enrichment of phenolic hydroxyl groups. 2,2-diphenyl-1-picrylhydrazyl (DPPH), 2,2′-azino-bis­(3-ethylbenzothiazoline-6-sulfonic acid) (ABTS), and ferric-reducing antioxidant power (FRAP) assays confirmed that lignin fractions with intermediate molecular weights and lower carbohydrate content exhibited stronger radical scavenging capacity than low-molecular-weight fractions with higher sugar levels.^15^ Zhao et al. demonstrated that both unmodified and grafted lignin with *p*-aminodiphenylamine improved aging properties compared to neat SBR. The chemically grafted lignin exhibited enhanced antiaging properties and better tensile strength retention, particularly at higher loadings.^16^

In this work, we utilized a sequential solvent fractionation process using a gradient of water/ethanol solution to treat four types of commercial lignin products, aiming to illustrate the correlations of the molecular weight, chemical composition, and antioxidation efficiency associated with the origin and treatment process of lignin. Among the four lignin products, the enzymatically hydrolyzed lignin (EHL) and its first fraction were chosen for further compounding with SSBR/silica due to their higher fraction yield and higher antioxidant properties. The impact of EHL lignin and its first fraction on silica dispersion, reinforcement, and antioxidative performance of SSBR/silica composites was investigated and compared to conventional 6PPD antioxidant-containing composites.

## 2 Results and Discussion

### 2.1 Lignin Fractionation and Characterization

Four commercial lignin products are selected in this study: kraft softwood lignin, UPM hardwood lignin, enzymatically hydrolyzed lignin, and Indulin AT kraft pine lignin, referred to as KL F0, UPM F0, EHL F0, and IAT F0, respectively. The FT-IR spectra of the raw lignin samples (F0) are shown in Figure 1 to compare the contributions of the G and S units in the different lignin samples. The overall spectrum is presented in Figure 1a, while the local spectral regions are highlighted in Figure 1b. The absorption bands at 1120 cm^–1^ (S ring breathing) and 1326 cm^–1^ (S and G ring condensed) confirmed the hardwood-based nature of UPM F0. Furthermore, the band at 1270 cm^–1^ originating from G ring breathing with C=O stretching was not apparent, while the peak was observed for the other three products. Moreover, the band at 1030 cm^–1^ from G units was observed for the four lignin samples.^17,18^ The intensity of the band of C=O became more pronounced in the F1 samples, except for UPM F1, as seen in Figure S1.

**Figure 1:**
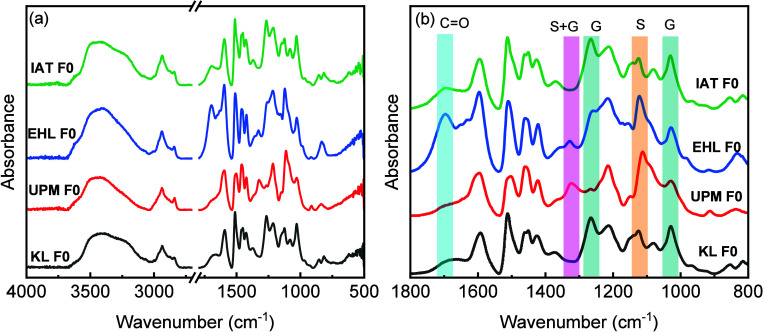
(a) FT-IR spectra of different lignin raw materials (KL F0, UPM F0, EHL F0, and IAT F0) and (b) local spectral region highlighting characteristic absorbance bands.

The molecular weights and dispersities of each fraction were characterized by gel permeation chromatography (GPC), as shown in Table 1 and Figure 2c-f. The overall fractionation procedure is schematically illustrated in Figure 2a. The first fraction (F1) from the four types of lignin have the lowest number- averaged molecular weight (*M*_n_), weight-averaged molecular weight (*M*_w_), and polydispersity index (PDI) as compared to the original lignin F0 and other fractions. The UPM F0 and its third fraction (UPM F3) exhibited poor solubility in dimethylformamide (DMF); hence, their *M*_n_ and *M*_w_ could not be determined using the same method. UPM F1 had the lowest *M*_W_ and PDI, as well as the lowest yield (6%), as seen in Figure 2. The second fraction of UPM (UPM F2) has the highest *M*_W_ and PDI values, as seen in Table 1. EHL F1 and IAT F1 showed higher yields of approximately 44 and 32%, respectively. The higher solubility of EHL in the ethanol/water solution is likely attributed to the presence of polar C=O groups, particularly carboxylic acids, and the presence of these groups is more evident in EHL F1 after fractionation, as confirmed by FT-IR analysis (see Figure S1).

**Table 1:** GPC Results of Four Lignin and Their Three Fractions^a^

code	***M***_**n**_ (g/m)	***M***_**w**_ (g/m)	PDI	code	***M***_**n**_ (g/m)	***M***_**w**_ (g/m)	PDI
KL F0	3300	6000	1.8	EHL F0	3030	5090	1.68
KL F1	2400	3280	1.37	EHL F1	2655	3790	1.42
KL F2	3258	5380	1.65	EHL F2	4040	7340	1.81
KL F3	5363	9970	1.85	EHL F3	4970	9830	1.97
UPM F0				IAT F0	3320	5850	1.76
UPM F1	2120	2700	1.27	IAT F1	2200	3080	1.40
UPM F2	5660	13960	2.46	IAT F2	3900	6330	1.62
UPM F3				IAT F3	6060	10,730	1.77

a GPC was performed using DMF with NH_4_BF_4_ buffer, and the MW was determined using narrow calibration with a poly­(methyl methacrylate) PMMA standard.

**Figure 2:**
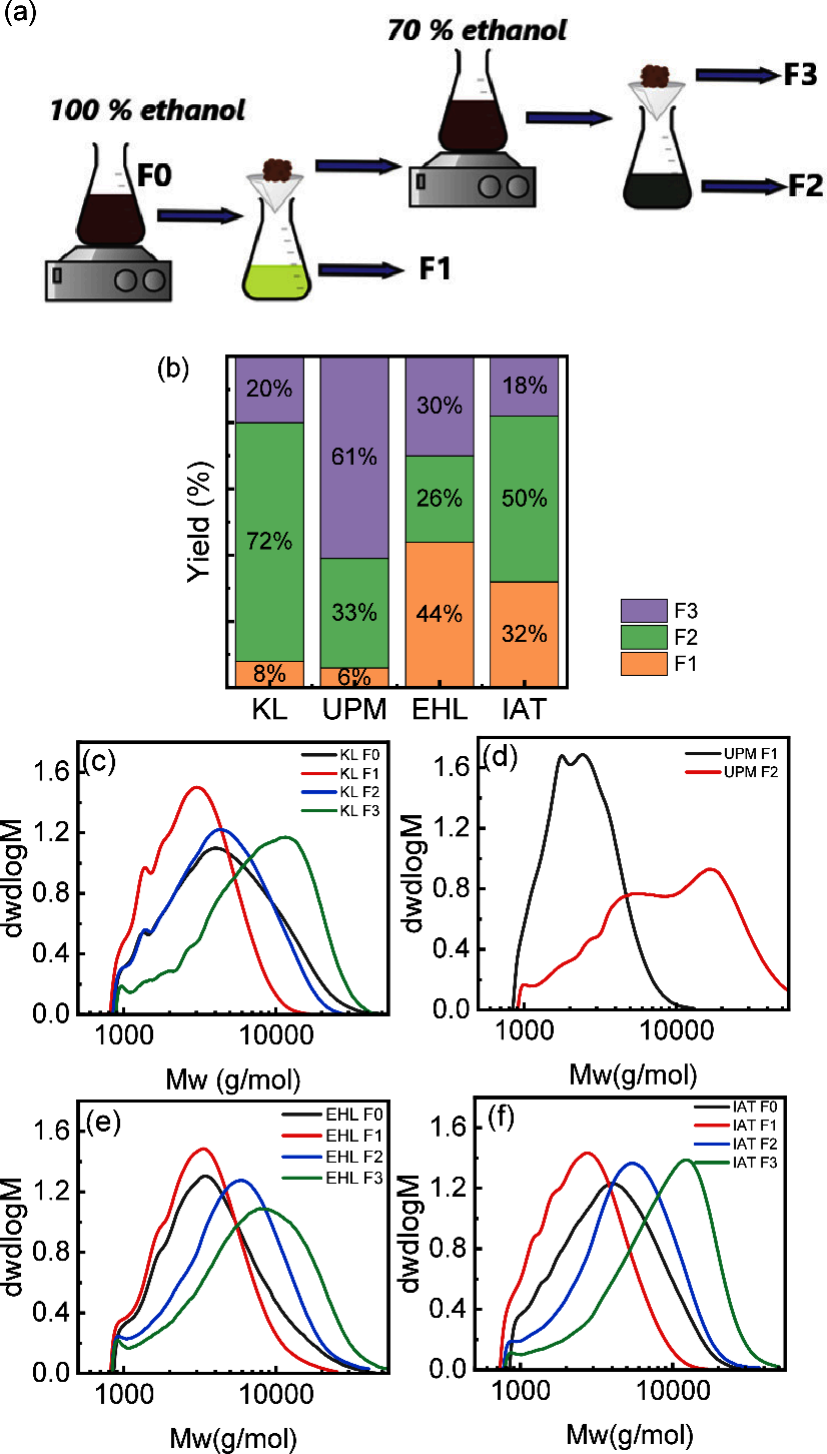
(a) Schematic representation of the stepwise fractionation process used to obtain lignin fractions F0–F3. (b) Yield of different fractions. The molecular weight distribution of fractions: (c) KL, (d) UPM, (e) EHL, and (f) IAT.

The total phenolic hydroxyl group content (phOH) was quantified for all samples except the third fractions (F3). Among all samples, F1 has the highest phOH, which is attributed to its lower *M*_w_, and thus higher ratio of functional groups, as seen in Figure 3a. F2 has a lower phOH . All fractions from EHL showed a lower than other grades. Figure 3b shows that the total phOH amounts from EHL F0 to F1 are close to each other, as approximately 44% of the F1 fraction was extracted from the F0.

**Figure 3:**
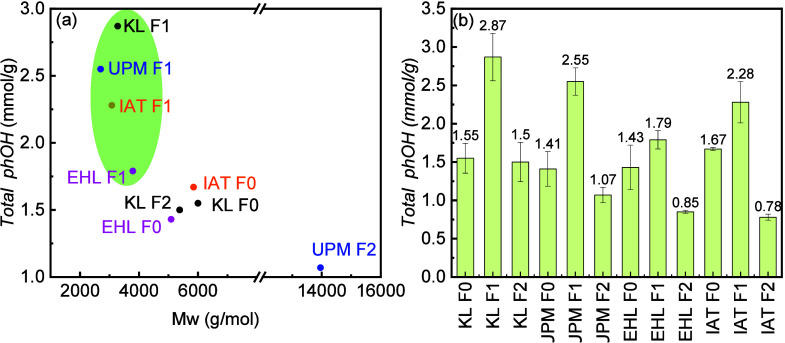
(a) Relationship between total phenolic hydroxyl group content (phOH) and *M*_w_ and (b) the total phenolic hydroxyl group content (phOH) of lignin fractions.

The radical scavenging activity (RSA) of the lignin fractions was determined by using UV–vis spectroscopy. Figure 4a illustrates that an increase in KL F0 concentration resulted in a drop in the absorbance value and an increase in RSA. The local maximum value of the DPPH blank was at 515 nm. A leftward shift was observed with an increase in concentration. As shown in Figure 4b, RSA values of F1 are also higher than those of their other fractions, complementary to the phenolic hydroxyl group content determination. The RSA values of EHL F0 and F1 were similar, showing 25.82 and 27.6%, respectively, in consistent with the result of their similar phenolic hydroxyl group contents, 1.43 and 1.79 mmol/g, respectively.

**Figure 4:**
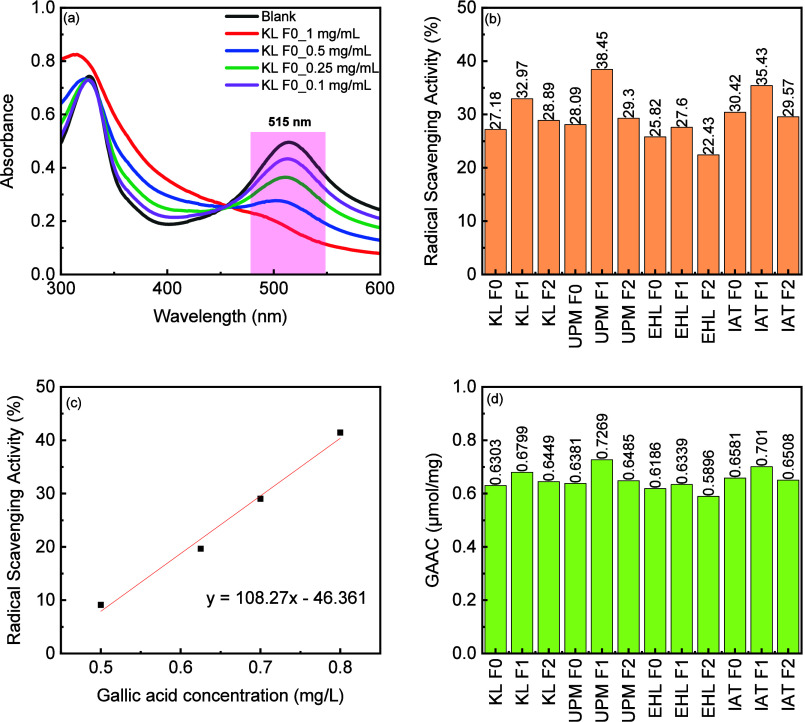
(a) UV–vis spectra of the DPPH blank and KL F0 at different concentrations, (b) RSA of lignin fractions, (c) standard curve of GA, and (d) GAACs of lignin fractions.

The scavenging activity was further cross-referenced to that of standard gallic acid (GA). GA with different concentrations was dropped in DPPH solution to prepare a calibration curve (Figure 4c) (*R* ^2^ = 0.98) and determine the antioxidant capacity of the lignin fractions. The greatest antioxidant capacity value, according to GAACs shown in Figure 4d, was 0.7269 for UPM F1, while the lowest antioxidant capacity was 0.6339 for EHL F1.

### 2.2 SSBR/Silica Composites Containing Lignin

#### 2.2.1 Large Amplitude Oscillatory Shear (LAOS) Analysis of SSBR Compounds and Vulcanizates

EHL F0 and F1 were selected to incorporate into SSBR/silica compounds due to the higher fractionation yield to allow rubber processing at scale. The effects of EHL F0 and F1 on silica dispersion in SSBR compounds were investigated by evaluating the nonlinear viscoelastic behavior using large amplitude oscillatory shear rheology (LAOS). Figure 5 displays the strain sweep results of the rubber compounds (a–c) and the vulcanizates (d–f) in the range of γ_0_ = 0.07–500 % at 60 °C and 1 Hz.

**Figure 5:**
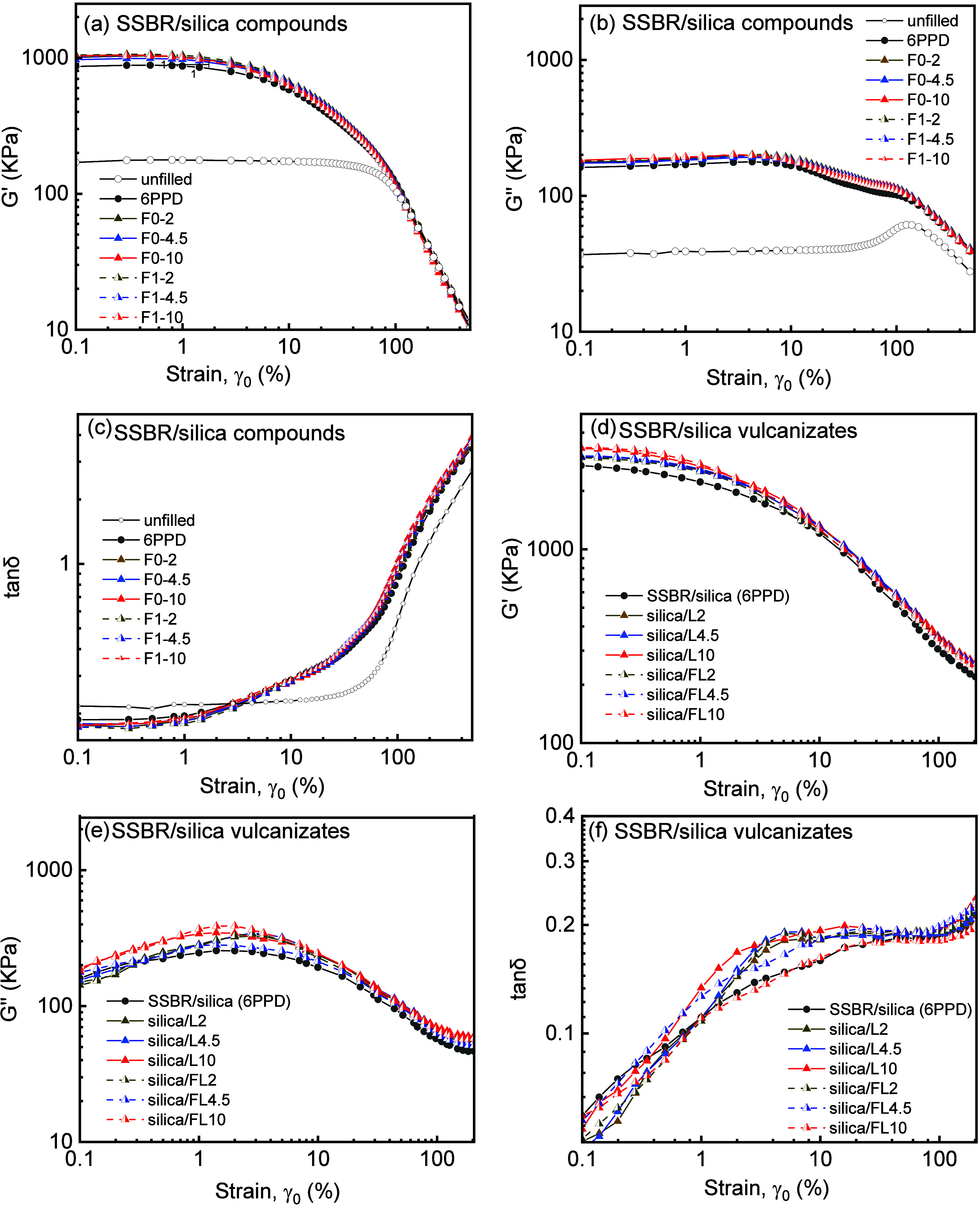
Strain sweep results of SSBR/silica composites at 1 Hz and 60 °C, (a) *G*′ vs γ_0_ of compounds, (b) *G*″ vs γ_0_ of compounds and (c) tan δ vs γ_0_ of compounds; and (d) *G*′ vs γ_0_, (e) *G*″ vs γ_0_ of vulcanizates, and (f) tan δ vs γ_0_ of vulcanizates.

As shown in Figure 5a–c, the uncured rubber compounds exhibited typical strain-softening behavior at γ_0_ > 1 %, i.e., the Payne effect. In the low and medium strain amplitudes, 1 < γ_0_ < 50%, the filled SSBR compounds exhibited higher storage moduli (*G*′) and loss moduli (*G*″) as compared to the unfilled SSBR. A weak *G*″ overshoot between 1.4 < γ_0_ < 7.3% was observed in the filled SSBR compounds and was ascribed to energy dissipation caused by interparticle frictions with strain amplitude.^19^ In contrast, for the unfilled sample, only one *G*″ overshoot was observed at 58%, associated with the second *G*″ overshoot observed in the filled SSBR compounds, which is attributed to energy dissipation by polymer chain motion.^20^ The effect of filler is also reflected on the loss factor (tan δ). A lower plateau at γ_0_ < 2.8% suggested constrained polymer chain motion by the fillers. As γ_0_ increases, the filler network is disrupted and results in a higher tan δ.

Herein, a characteristic critical strain amplitude (γ_c_) is defined as the transition point by extrapolating from the linear and nonlinear regimes in log *G*′ vs log γ to further quantify the amplitude sweep test. After vulcanization, the synergetic effect of the chemically crosslinked network and the filler–filler interactions contributes to the smaller γ_c_ and broad overshoot of *G*″ along with increasing tan δ even at small strain amplitude.^19,21,22^

The incorporation of 2∼10 phr lignin increased Δ*G*′ from ∼2640 to 3000 kPa, while γ_c_ varied subtly, as shown in Table 2. To further visualize the effect of lignin on the nonlinear regime of SSBR/silica compounds, Fourier-transform rheology analysis was performed to compare the relative intensity of the third harmonic to the first harmonic (*I*_3/1_).^23,24^ The *I*_3/1_ vs γ_0_ curve of the filled SSBR compounds in Figure 6a exhibited a double-arc shape, indicating two distinct nonlinear responses across the measured γ_0_. The local minimum (γ_min_) in between was defined as the transition from the medium amplitude oscillatory regime (MAOS) to the large amplitude oscillatory regime (LAOS), which correspond to the *G*′(γ_0_) decay and *G*″ overshoot by filler–filler network deformation and breakdown (the first arc shape), followed by polymer network disentanglement in the LAOS regime as aforementioned. Increasing content of lignin results in larger *I*_3/1_ in the MAOS regime and reduced γ_min_, as seen in Figure 6b. This indicates that higher loading of lignin increased the nonlinearity of the composites by a denser network and thus earlier breakdown under smaller strain. This also highlights the importance of Fourier-transform rheology to signify such differences, where only a subtle change in Δ*G*′ was observed in the conventional log *G*′ vs log γ plot.

**Table 2:** Rheometric Characteristics of SSBR Composites

	compounds	vulcanizates
samples	Δ*G*′ (*G*_0.1_′ – *G*_100_′) (KPa)	
unfilled SSBR	68	152
SSBR/silica/6PPD	853	2400
SSBR/silica/F0-2	996	2645
SSBR/silica/F0-4.5	959	2674
SSBR/silica/L10	1026	2960
SSBR/silica/F1-2	1036	2620
SSBR/silica/F1-4.5	998	2652
SSBR/silica/F1-10	1021	3007

**Figure 6:**
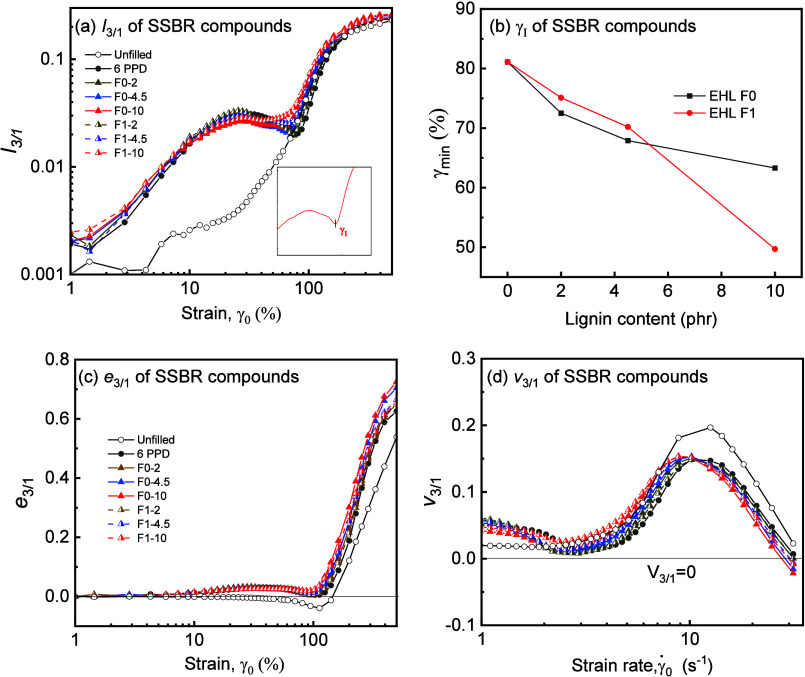
(a) *I*_3/1_ ∼ γ_0_, (b) γ_min_ ∼ lignin content, and (c) *e*_3/1_ ∼ γ_0_ and (d) *v*_3/1_ of uncured SSBR composites ∼ γ̇_0_ at 1 Hz and 60 °C for uncured SSBR compounds.

The nonlinear behavior decoupled the *I*_3/1_ ∼ γ_0_ into the elastic and viscous components, characterized by Chebyshev polynomials parameters, *e*_1_, *e*_3_, *v*_1_, and *v*_3_.^23,25^ Analogously, the relative components $e_{3/1}=\frac{e_{3}}{e_{1}}$ and $v_{3/1}=\frac{v_{3}}{v_{1}}$ shown in Figure 6c,d were used to quantify the intracycle elastic and viscous nonlinearities, respectively.^26^ As shown in Figure 6c, the unfilled SSBR first gradually decreased in negative *e*_3/1_ until reaching a minimum at γ_0_ of 140 %, indicating intracycle strain softening triggered by conformation change of the entangled network;^27^ then, it exhibited strain stiffening by deformation-induced chain orientation, where *e*_3/1_ increasing rapidly at larger γ_0_.^28^ The *e*_3/1_ of all SSBR compounds containing silica and lignin remained positive at γ_0_ < 100%, suggesting strain hardening due to the constraint by the filler network. At larger γ_0_, the filled composites exhibited enhanced strain stiffening as the unfilled SSBR. This was further confirmed by the elastic Bowditch–Lissajous plot, with the upturn of the stress signal at the large strain regime (γ_0_ of 255%) as the indication of strain hardening in Figure S4a.

SSBR compounds showed intracycle strain thickening with *v*_3/1_ > 0 across the γ̇_0_ of 0.0628–31.42 s^–1^(corresponding to the γ_0_ of 1–500 %) except at high γ̇_0_, as seen in Figure 6d. The *v*_3/1_ of the unfilled SSBR compounds stayed steady at low γ̇_0_ (<2.72 s^–1^, corresponding to the γ_0_ of 43.35%), while exhibited a pronounced peak at the γ̇_0_ of 12.57 s^–1^ (corresponding to the γ_0_ of 200%), indicating enhanced strain thickening from medium to large γ̇_0_ range (as well as the medium to large strain amplitude range); afterward, the strain thickening behavior weakened at higher γ̇_0_.^27^ With the filler network of lignin and silica in the filled compounds, the strain thickening behavior also presented and intensified with increasing lignin content at low γ̇_0_, which was associated with constrained polymer chain rearrangement and disentanglement.^25^

#### 2.2.2 Crosslinking Density and Mechanical Properties

Curing curves of SSBR/silica composites at 160 °C are shown in Figure S2a, and the characteristics, including the maximum torque (*S*′_max_), the minimum torque (*S*′_min_), and the scorch time (the end point of the induction period, *t*_s2_), and the difference between *S*′_max_ and *S*′_min_ (Δ*S*), are summarized in Table S1. The addition of lignin EHL F0 and F1 reduced the *S*′_max_ at low contents, followed by an increase at higher loadings. and the conversion rate from the 1st derivative was also lowered when lignin was introduced (Figure S2b,c).^29^

The SSBR compounds were vulcanized at 160 °C under a pressure of 12 MPa according to the optimum curing time (*t*_90_) in Table S1. The crosslink density (*V*_c_) of SSBR composites increased by 9.3 and 7.4%, respectively, at 2 phr EHL F0 or F1 compared to the silica-only filled benchmark, as seen in Figure 7a. Then, *V*_c_ approached a constant as the lignin content increased. In the EHL F1 composite, *V*_c_ plateaued at 0.573 mmol/cm^3^ with 4.5 and 10 phr EHL F1. It was postulated that the initial introduction of lignin enhanced the bound rubber content; as lignin content further increases, the aggregation of lignin does not generate a new interface to enhance bound rubber content, and the crosslink density reached a maximum.

**Figure 7:**
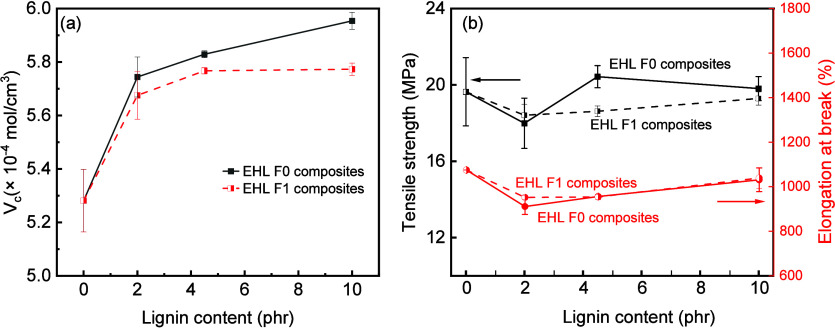
(a) *V*_c_ of SSBR/silica composites to the lignin content and (b) tensile strength and elongation at breakof SSBR vulcanizates.

The tensile strength and elongation at break of the control sample SSBR/silica (6PPD) composite were 19.64 ± 1.79 MPa and 1075 ± 2%, respectively. As shown in Figure 7b, by replacing 6PPD with EHL F0 or F1, the tensile strength and elongation at break of SSBR/silica composites decreased slightly at 2 phr F0 or F1, then increased to 19.81 ± 0.35 and 19.29 ± 0.63 MPa at 10 phr loading, respectively. This shows that lignin (2–10 phr) has a limited reinforcement effect on the rubber composites.

### 2.3 Effect of Lignin on the Thermo-Oxidation of SSBR Composites

As discussed in Section 2.1, lignin exhibits free radical scavenging capability to mitigate radical degradation of polymer molecules. The thermo-oxidation of SBR primarily involves the formation of hydroxyl and carboxyl groups as a result of oxygen attacking the butadiene units, which is then followed by chain scission.^30–32^ The thermo-oxidative resistance of SSBR composites containing F0 and F1 was evaluated by the oxidation induction time (OIT) test at 210 °C and the accelerated aging test at 120 °C. The OIT results of SSBR composites are shown in Figure 8, where heat generated by exothermic thermo-oxidative degradation was monitored and characterized by the time at maximum heat flow. Without any antioxidants, the oxidation induction time was 0.01 min in the SSBR/silica composite. The OITs of SSBR/silica composites containing 6PPD or EHL were delayed, indicating their free radical scavenging effect. The OITs of SSBR/silica composites containing 4.5–10 phr EHL F0 or F1 varied between 10.28–11.03 min and were shorter than that of SSBR/silica (6PPD) (34.88 min), indicating less effective F0 and F1 in free radical scavenging than 6PPD in the O_2_ environment.

**Figure 8:**
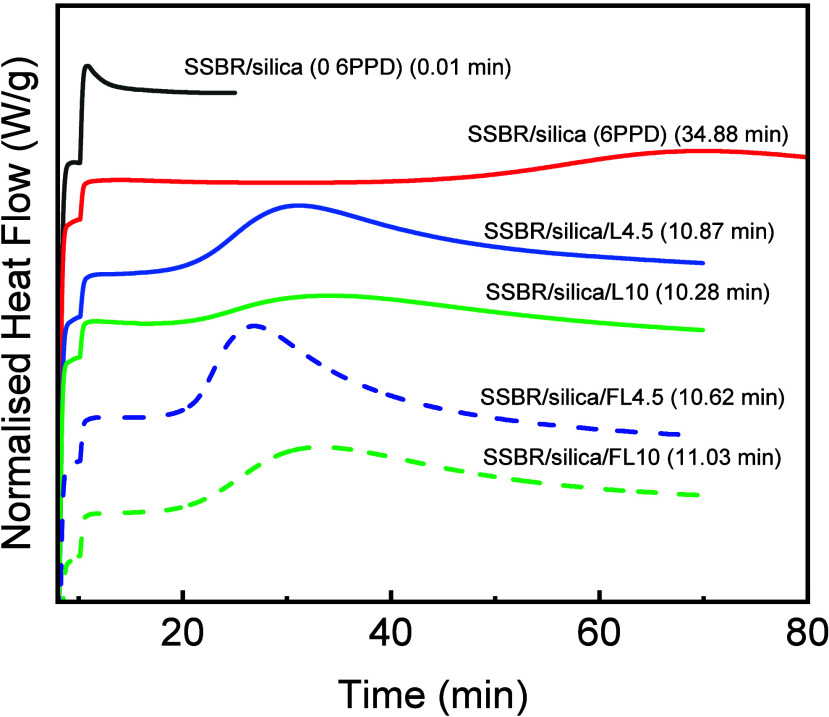
OIT results of SSBR composites at 210 °C.

Accelerated thermal aging test was conducted for the rubber composites at 120 °C for 12 days. The mechanical properties were evaluated every 4 days to study the effects of temperature and aging time on the thermo-oxidative degradation process. The control measurements were conducted at ambient temperature. As shown in Figure 9a,b, the tensile strength and the elongation at break of all composites deteriorated to different extents along with the aging time. Along with the free radical propagation mechanism, the crosslinking reaction could also be triggered at elevated temperatures.^33^ After aging for 20 days at 120 °C, the tensile strength with 2 phr 6PPD has the highest tensile strength of 7.97 ± 1.12 MPa but the lowest elongation at break of 82 ± 7%, while the tensile strength of SSBR vulcanizates containing lignin F0 or F1 was kept in the range of 6.06 ± 1.2 and 7.94 ± 0.24 MPa, with the elongation at break of 95 and 124%, respectively. The relative tensile strength and the elongation at break compared to fresh samples over aging time are shown in Figure 9c,d. For 2 phr 6PPD, the tensile strength of the SSBR/silica (6PPD) was 76.3 ± 4.0% after 4 days and decreased to 40.6 ± 5.7% after 20 days. Compared with lignin-filled samples, 2 phr F0 had the highest retention of tensile strength with a value of 44.10 ± 1.3% after 20 days, whereas 2 phr F1 (fractionated lignin) was comparable to 6PPD. The equivalent or even better aging resistance by lignin, as opposed to the longer inhibition time by 6PPD in the OIT test, was plausibly attributed to (i) distinct testing methods, where the OIT test measures heat generation from exothermic thermo-oxidative degradation, which primarily reflects chemical reactions, whereas the aging test assesses mechanical properties, which are influenced by a broader range of factors (e.g., composite structures, filler interactions, and dispersion); and (ii) testing conditions: OIT is conducted at 210 °C, much higher than the 120 °C used in the aging test. At this elevated temperature, side reactions, such as peroxide reactivation and lignin’s self-degradation, also take place, whereas these species remain inert at the lower temperature used in the aging test.

**Figure 9:**
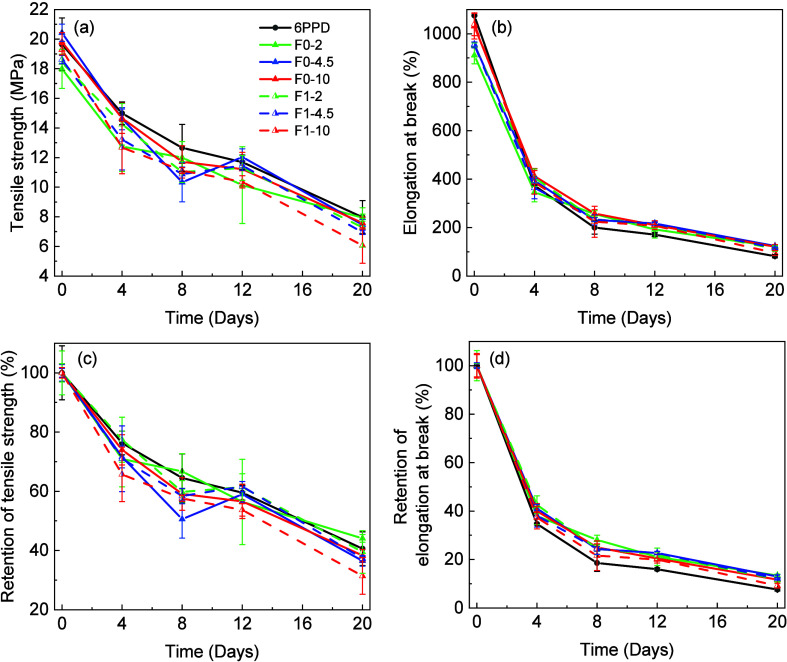
(a) Tensile strength, (b) elongation at break, and retention ratio of (c) tensile strength and (d) elongation at break of the SSBR composites.

## 3 Conclusions

This study systematically evaluated the chemical composition and antioxidant functionality of lignin and its fractionations through a sequential extraction process using a gradient mixture of ethanol and water. KL F1 and UPM F1 exhibited the highest total phOH content and the greatest radical scavenging activity (RSA) of 38.45% but the lowest yields (8 and 6%). EHL F1 had a moderate RSA of 27.6% among the four lignin grades but the highest yield (44%). Then, the effects of EHL F0 and F1 on the mechanical and thermo-oxidative resistance of silica-filled SSBR composites were evaluated at a loading of 2∼10 phr. The incorporation of EHL F0 and F1 did not significantly increase the Payne effect and viscoelastic nonlinearities of SSBR compounds at concentrations below 10 phr. SSBR/silica/F0-2 and SSBR/silica/F1-2 exhibited comparable thermo-oxidative resistance to SSBR/silica (6PPD), with tensile strength retentions of 44.10 ± 1.3 and 31.4 ± 6.2%, respectively, after accelerated aging at 120 °C for 20 days. This study shows that <10 phr lignin can be used as a bioavailable and less hazardous antioxidant to enhance the long-term thermo-oxidative resistance of SSBR/silica composites without affecting the nonlinear dynamic properties.

## 4 Experimental Section

### 4.1 Materials

Solution-polymerized butadiene-styrene rubber (SSBR, grade Buna® VSL 4526-2 HM, extended with 37.5 phr or 27.3 wt % TDAE oil) was provided by ARLANXEO Co., Ltd. Four types of commercial lignin products were selected: Kraft lignin (softwood, Sigma-Aldrich UK), UPM lignin (hardwood, X40 grade, UPM Co., Ltd., Germany), EHL (enzymatically hydrolysis lignin originated from corncob waste, Shandong Longlive Bio-Technology Co., Ltd., China), and Indulin AT (kraft pine lignin, Ingevity Co., Ltd., UK), referred to as KL, UPM, EHL, and IAT respectively. The silica (grade GR7000) was purchased from Evonik Industries Co., Ltd. Other additives and solvents, including zinc oxide (ZnO), stearic acid, sulfur, diphenylguanidine (DPG), *N*-*tert*-butyl-2-benzothiazole sulfenamide (TBBS), anhydrous ethanol (purity ≥99.8 %), 6PPD (purity ≥99.8 %), tetrahydrofuran (THF, purity ≥99.9 %), toluene (purity ≥99.9 %), cyclohexane (purity ≥99.9 %), DPPH, ethyl alcohol (purity ≥99.9%, suitable for HPLC), dimethyl sulfoxide (DMSO**,** ACS reagent, ≥99.9%), sodium hydroxide (NaOH, ACS reagent, ≥97.0%, pellets), and gallic acid (GA, *M*_w_: 170.12 g/mol) were purchased from Sigma-Aldrich, UK. Acetate buffer solution (pH 6) was purchased from Fisher Chemical.

### 4.2 Preparation

#### 4.2.1 Lignin Fractionation

The commercial lignin products were first treated by ball milling using PM100 (Retsch) to further reduce the particle size. Briefly, 10 g lignin powders were milled with stainless steel balls (5 mm diameter, 100 g) in an 80 mL milling jar for 2 h and subsequently milled with stainless steel balls (2 mm diameter, 200 g) for 2 h at a constant speed of 450 rpm. An interval of 10 min was allowed every 15 min of milling to avoid heat buildup. The ball-milled lignin was denoted as F0.

The F0 lignin was first dissolved in ethanol (100%), and the soluble and insoluble parts were separated by vacuum filtration, followed by rotary evaporation at 40 °C. The soluble part was designated as F1. The remaining insoluble part was further dissolved in ethanol/water (70/30 v/v) at room temperature, and the soluble part in the cosolvent was separated, collected, and dried, designated as F2. The remaining insoluble part was designated as part F3. The procedure is also illustrated in Figure 2a.

#### 4.2.2 Lignin Fraction Characterization

FT-IR ATR spectra were collected on a Bruker Vertex 70 PTIR spectrometer at a resolution of 4 cm^–1^, with 16 scans at room temperature. GPC was performed using an Agilent Infinity II MSDS equipped with a differential refractive index detector, a viscometer, and a light scattering detector; DMF containing 5 mM NH_4_BF_4_ as the eluent; and PMMA as a narrow calibration standard.

phOH was measured following the method described by Serrano et al.,^34^ using an Agilent Cary 60 UV–Vis spectrophotometer with the equation 1:^35^

$$\mathrm{Total}\;\mathrm{phOH}=0.425\times A_{300\mathrm{nm}}(\mathrm{NaOH})+0.182\times A_{350\mathrm{nm}}(\mathrm{NaOH})\times a$$ 1

where *A* is the absorbance, and *a* is the correction term (L·g^–1^ ·cm^–1^).

*A*_300 nm_ indicates the presence of free phOH groups, while the conjugated absorbance, particularly that of carbonyl groups (C=O), is related to *A*_350 nm_.^35^

To determine the antioxidant activity of the raw materials and their fractions, a DPPH assay was conducted using the methods given by Aadil et al.^36^ and Gordobil et al.^37^

An initial optimization step was carried out using only KL to determine an appropriate lignin concentration for the DPPH assay. KL was dissolved in DMSO with following concentrations: 0.1, 0.25, 0.5, and 1 mg/mL. Then, 0.1 mL of KL solutions were mixed with 3.9 mL of DPPH solution (18.28 mg/L) and shaken. Afterward, the solutions were put in a dark environment for 30 min, and then the UV–vis spectra were recorded. A characteristic peak for the DPPH blank solution appears at around 515 nm. The lignin solution with a concentration of 0.25 mg/mL was finally chosen for mixing with DPPH solution for further analysis. Furthermore, GA, a well-known antioxidant, was used to construct a calibration curve that served as a reference to compare the antioxidant capacity of lignin samples. Radical scavenging activities (RSAs) and gallic acid antioxidant capacities (GAACs) were calculated by using the equations 2–4:

$$\mathrm{RSA}(\%)=(1-(A_{\mathrm{sample}}/A_{\mathrm{blank}}))\times 100$$ 2

$$C_{\mathrm{GE}}\;[\mu g/\mathrm{mL}]=\frac{\mathrm{RSA}-b_{\mathrm{cal}}}{m_{\mathrm{cal}}}$$ 3

$$\mathrm{GAAC}\;[\mu \mathrm{mol}/\mathrm{mg}]=\frac{c_{\mathrm{GE}}\times \frac{1}{M_{\mathrm{GA}}}}{c_{\mathrm{lignin}}}$$ 4

where *C*_GE_ is the GA equivalent, *M*_GA_ is the molarity of GA, and *c*_lignin_ is the lignin concentration.

#### 4.2.3 Preparation of SSBR/Silica Composites and Characterization

SSBR/silica compounds containing EHL F0 and its F1 were prepared following the formulations specified in Table 3. The SSBR composites containing 2–10 phr EHL F0 and F1 were denoted as SSBR/silica/F0-2–10 and SSBR/silica/F1-2–10, respectively. The unfilled SSBR compound and silica-filled SSBR containing 6PPD as the antioxidant were prepared as control samples, denoted as unfilled SSBR and SSBR/silica, respectively. A two-step compounding process was adopted for preparing the composites. For the first step, all the materials except the curing agent and accelerators (sulfur, TBBS, and DPG) were compounded using a Brabender internal mixer B50 at 80 to 120 °C, 60 rpm for 10 min, and then further processed using a twin-roll mill 5 times at 40 °C. For the second step, the master mixture was further compounded with the curing agent and accelerators at 80 °C for 6 min and passed through the two-roll mill 5 times at 40 °C again.

**Table 3:** Formulations of SSBR/Silica/Lignin Compounds (in phr)

sample code	unfilled SSBR	SSBR/silica	SSBR/silica/(F0 or F1)
SSBR	100	100	100
silica	0	50	50
EHL F0 or F1	0	0	2–10
6PPD	2.0	2.0	0
ZnO	5.0	5.0	5.0
stearic acid	2.0	2.0	2.0
sulfur	1.5	1.5	1.5
TBBS	1.5	1.5	1.5
DPG	2.0	2.0	2.0

The crosslink density was determined by an equilibrium swelling test in toluene based on the Flory–Rehner equation.^38^ The equilibrium swelling experiment was conducted by immersing the cured rubber samples in toluene at room temperature for 72 h. The samples were immediately weighed after the solvent was wiped off quickly from the sample surface using filter paper and dried in a vacuum oven at 60 °C until a constant weight was achieved. The crosslink density of each sample was presented as the average of three measurements. The elastically active network chain density can be calculated using the Flory–Rehner equation 5:

$$V_{e}=\frac{-(\ln(1-V_{r})+V_{r}+\chi V_{r}^{2})}{V_{s}\left(V_{r}^{1/3}-\frac{V_{r}}{2}\right)}$$ 5

where χ is the Flory–Huggins polymer solvent interaction parameter (0.446 for SBR and toluene), and *V*_s_ is the molar volume of the solvent (106.5 cm^3^/mol for toluene). The volume fraction (*V*_r_) was calculated using the equation 6:

$$V_{r}=\frac{\frac{m_{2}-m_{0}\phi }{\rho_{r}}}{\frac{m_{2}-m_{0}\phi }{\rho_{r}}+\frac{m_{1}-m_{0}}{\rho_{s}}}$$ 6

where *m*_0_ is the sample mass before swelling; *m*_1_ and *m*_2_ are the weights of the swollen sample before and after drying, respectively; ϕ is the weight fraction of the insoluble components; and ρ_r_ (1.12 g/cm^3^) and ρ_s_ (0.87 g/cm^3^) are the densities of the rubber and solvent, respectively.

The curing behavior of compounded rubbers was evaluated using a D-RPA 3000 Dynamic Rubber Process Analyzer (Montech) at 1.67 Hz with an angle of 0.5° at 160 °C. The curing torque (*S*′) against time was recorded, and the curing characteristics were calculated. Strain sweep tests were conducted in the range of 0.07–500% at 1 Hz at 60 °C for the uncured rubber compound and in the range of 0.07–200% for the cured rubber composites.

Tensile testing was performed at 200 mm/min at room temperature, using a Shimadzu Autograph AGS-X tester, according to ASTM D638-14 type V.

The accelerated aging test was performed according to ISO 00188-2023 (E) Rubber, volcanized or thermoplastic—accelerated aging and heat resistance tests. A Genlabprime oven with an accuracy of 0.6 °C and a stability variation of less than 0.5 °C was used for the experiment. The aging temperature was set as 120 °C. Tensile properties were evaluated after 0, 4, 8, 12, and 20 days, following ASTM D638-14 type V.

The oxidative induction time (OIT) measurement was performed using TA DSC25 according to ASTM D3895-19 to investigate the thermos-oxidative resistance. The program included the heating process at a rate of 20 °C/min to 210 °C, 5 min of equilibration of the sample at the temperature, and the isothermal process maintaining the aging temperature. N_2_ was pre-purged into the instrument for at least 5 min at a flow rate of 50 mL/min, the program was started until thermal equilibration, and the gas was changed into O_2_, purged at a flow rate of 50 mL/min until the first exothermic peak was detected. The development of the heat flow (normalized to the sample mass) dependent on isothermal time was recorded, and the onset of the first exothermic peak from the linear slope and the baseline was defined as the OIT.

## Supplementary Material



## Data Availability

The data that support the findings of this study are available from the corresponding author upon reasonable request.

## Notes

Additional experimental results; FT-IR spectra; torque (*S*′) vs. curing time of SSBR composites; curing characteristics; section morphology; elastic and Lissajous–Bowditch plots; and conversion rate equation used for the curing process (PDF)

## References

[1] Barana D. (2018). Lignin Based Functional Additives for Natural Rubber. ACS Sustainable Chem. Eng..

[2] Abid U. (2021). Potential applications
of polycarbohydrates, lignin, proteins, polyacids,
and other renewable materials for the formulation of green elastomers. Int. J. Biol. Macromol..

[3] Lu X., Gu X., Shi Y. (2022). A review on
lignin antioxidants: Their sources, isolations,
antioxidant activities and various applications. Int. J. Biol. Macromol..

[4] Roy K., Debnath S.C., Potiyaraj P. (2020). A Review on
Recent Trends and Future
Prospects of Lignin Based Green Rubber Composites. Journal of Polymers and the Environment.

[5] William, M. Compounding finely divided solids with butadiene-styrene synthetic rubber latices utilizing alkaline lignin solutions as dispersing agents. 1951, Google Patents.

[6] Yu P. (2016). A comprehensive
study on lignin as a green alternative of silica
in natural rubber composites. Polym. Test..

[7] Qiu J. (2023). Study on lignin amination
for lignin/SiO(2) nano-hybrids towards
sustainable natural rubber composites. Int.
J. Biol. Macromol..

[8] Hait S. (2021). Treasuring waste lignin
as superior reinforcing filler in high cis-polybutadiene
rubber: A direct comparative study with standard reinforcing silica
and carbon black. Journal of Cleaner Production.

[9] Hait S. (2024). Unlocking the potential
of lignin: Towards a sustainable solution
for tire rubber compound reinforcement. Journal
of Cleaner Production.

[10] Liu F. (2024). Improving Dispersion of Alkali Lignin in Natural Rubber
by Adopting
Graphene Oxide as a Carrier. ACS Applied Polymer
Materials.

[11] Murray G., Watson W. (1948). Lignin as a stabilizer for GR-S..

[12] Zhao M. (2024). Ozone resistance of
three natural antioxidants in solution polymerized
styrene-butadiene rubber—Molecular simulation and experimental
study. Polymer Degradation and Stability.

[13] Li K. (2024). Exploring the relationship
between lignin structure and antioxidant
property using lignin model compounds. Int.
J. Biol. Macromol..

[14] Xiao L. (2021). Study on the antioxidant
activity of lignin and its application performance
in SBS elastomer. Industrial & Engineering
Chemistry Research.

[15] Cheng X.-C. (2022). Sequential aqueous acetone fractionation and
characterization of
Brauns native lignin separated from Chinese quince fruit. Int. J. Biol. Macromol..

[16] Zhao S. (2021). Preparation of lignin-based
filling antioxidant and its application
in styrene-butadiene rubber. J. Appl. Polym.
Sci..

[17] Pandey K.K. (1999). A study
of chemical structure of soft and hardwood and wood polymers by FTIR
spectroscopy. J. Appl. Polym. Sci..

[18] Toscano G. (2022). FTIR spectroscopy for
determination of the raw materials used in
wood pellet production. Fuel.

[19] Yang R., Song Y., Zheng Q. (2017). Payne effect of silica-filled
styrene-butadiene
rubber. Polymer.

[20] Donley G.J., Singh P.K., Shetty A., Rogers S.A. (2020). Elucidating the G″
overshoot in soft materials with a yield transition via a time-resolved
experimental strain decomposition. Proc. Natl.
Acad. Sci. U. S. A..

[21] Song Y., Xu Z., Wang W., Zheng Q. (2021). Payne effect of carbon black filled
natural rubber nanocomposites: Influences of extraction, crosslinking,
and swelling. J. Rheol..

[22] Song Y. (2019). Rigid nanoparticles
promote the softening of rubber phase in filled
vulcanizates. Polymer.

[23] Nie S., Lacayo-Pineda J., Wilhelm M. (2019). Fourier-transform rheology of unvulcanized
styrene butadiene rubber filled with increasingly silanized silica. Soft Mater..

[24] Xia T. (2023). Effective and Fast-Screening Route to Evaluate Dynamic
Elastomer-Filler
Network Reversibility for Sustainable Rubber Composite Design. ACS Sustainable Chem. Eng..

[25] Fan X. (2020). Influences of chemical
crosslinking, physical associating, and filler
filling on nonlinear rheological responses of polyisoprene. J. Rheol..

[26] Hu Z. (2023). Strain softening of natural rubber composites filled with carbon
black and aramid fiber. J. Rheol..

[27] Lao T. (2024). Influences of non-rubber
components on the molecular network and
viscoelasticity of natural rubber gum. Polymer.

[28] Huang G. (2017). Strain Hardening Behavior
of Poly­(vinyl alcohol)/Borate Hydrogels. Macromolecules.

[29] Liu R. (2022). Comparative
study on the synergistic reinforcement of lignin between
carbon black/lignin and silica/lignin hybrid filled natural rubber
composites. Ind. Crops Prod..

[30] Guo L., Huang G., Zheng J., Li G. (2014). Thermal oxidative degradation
of styrene-butadiene rubber (SBR) studied by 2D correlation analysis
and kinetic analysis. J. Therm. Anal. Calorim..

[31] Rezig N., Bellahcene T., Aberkane M., Nait Abdelaziz M. (2020). Thermo-oxidative
ageing of a SBR rubber: effects on mechanical and chemical properties. J. Polym. Res..

[32] Zhao W. (2023). Recent progress in the
rubber antioxidants: A review. Polym. Degrad.
Stabil..

[33] Wang X., Yang K., Zhang P. (2022). Evaluation
of the aging coefficient
and the aging lifetime of carbon black-filled styrene-isoprene-butadiene
rubber after thermal-oxidative aging. Compos.
Sci. Technol..

[34] Serrano L. (2018). Fast, Easy, and Economical
Quantification of Lignin Phenolic Hydroxyl
Groups: Comparison with Classical Techniques. Energy Fuels.

[35] Gartner A., Gellerstedt G., Tamminen T. (1999). Determination of phenolic hydroxyl
groups in residual lignin using a modified UV-method. Nordic Pulp Paper Res. J..

[36] Aadil K.R. (2014). Free radical scavenging
activity and reducing power of Acacia nilotica
wood lignin. Int. J. Biol. Macromol..

[37] Gordobil O. (2018). Potential use of kraft
and organosolv lignins as a natural additive
for healthcare products. RSC Adv..

[38] Yu S. (2020). Effects of dynamic covalent
bond multiplicity on the performance
of vitrimeric elastomers. Journal of Materials
Chemistry A.

