# Supplementary material for: Effects of Lignin Fractionation on the Mechanical and Thermo-Oxidation Properties of SSBR/Silica Composites
Source: ACS Sustain Resour Manag. 2025 Nov 21;2(12):2471–81. doi: 10.1021/acssusresmgt.5c00496 (PMC12746543; doi:10.1021/acssusresmgt.5c00496)
Supplement: Supplementary file 1 [file rm5c00496_si_001.pdf]

Supporting Information

Effects of lignin fractionation on the mechanical and thermo-oxidation properties of SSBR/silica composites

Onur Nuri Arslan [1], Xiao Hu [1], Yanxi Shi[1], Haifeng Liu [2], Wai Hin Lee [1], Ming Li [2], Chaoying Wan*

[1] *International Institute for Nanocomposites Manufacturing (IINM), WMG, University of Warwick, UK, CV4 7AL*

[2] *Nanjing Tech University, Nanjing, Jiangsu, China, 211816*

Corresponding: Chaoying.wan@warwick.ac.uk

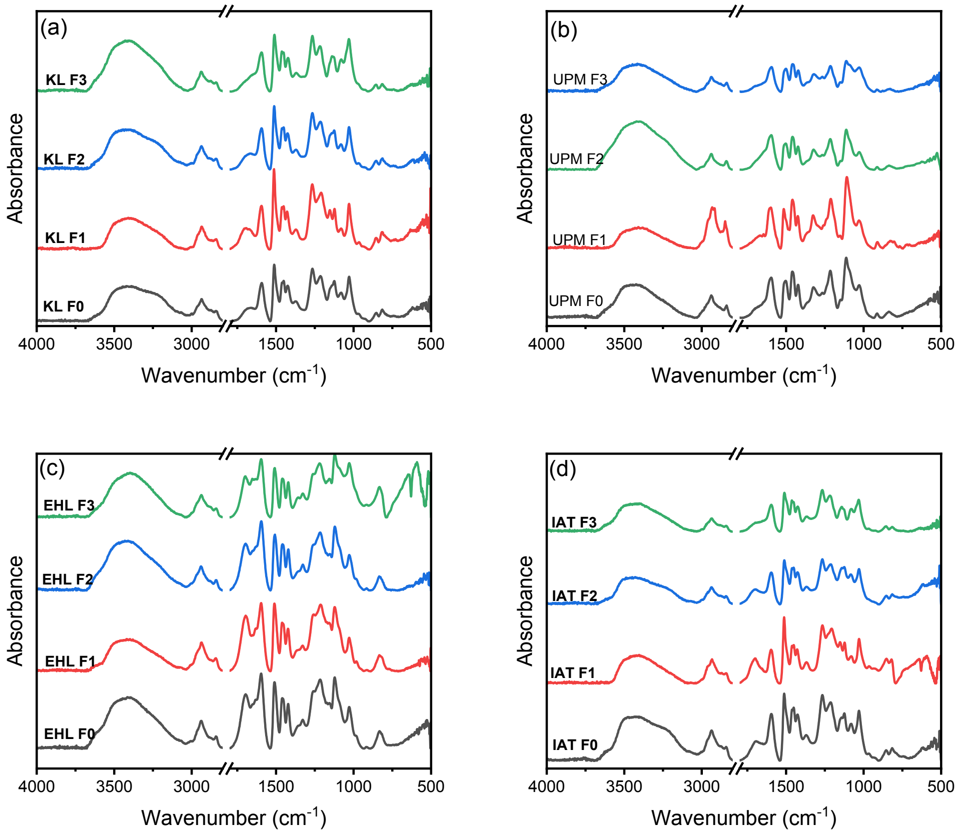

Figure S1. FTIR Spectra of Lignin Fractions: (a) KL, (b) UPM, (c) EHL, (d) IAT.

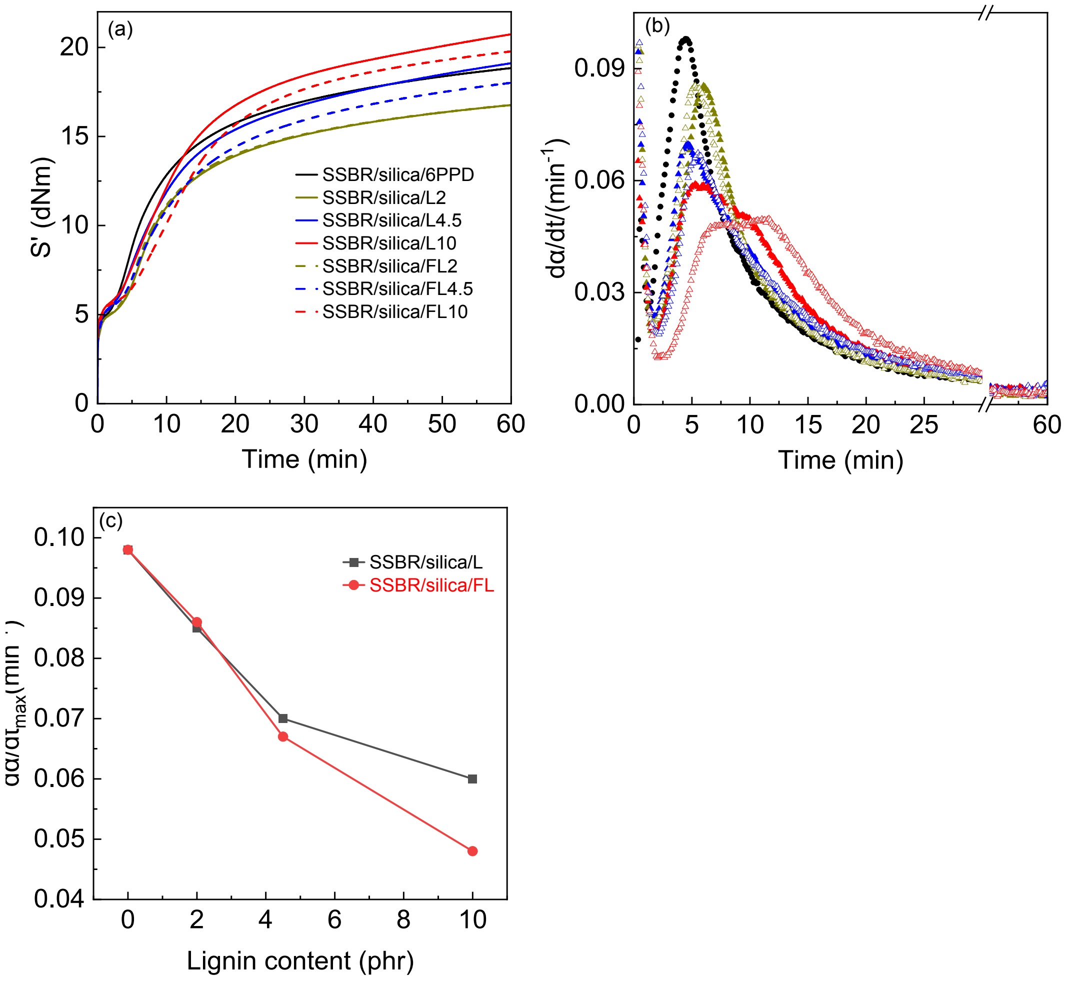

Figure S2 (a) The torque (S') vs. the curing time of SSBR composites at 160 °C for 60 min, (b) the development of the minimum torque (S'_{min}) and the difference between maximum torque (S'_{max}) and S'_{min} (ΔS) vs. the lignin content, (c) the relationship of the conversion rate ($d\alpha/dt$) of SSBR/silica composites to the time, and (d) the relationship of maximum $d\alpha/dt$ ($d\alpha/dt_{max}$)

Table S1 Curing characteristics, and mechanical properties of SSBR composites

Samples	S'_{min} (dNm)	S'_{max} (dNm)	ΔS (dNm)	t_{s2} (min)	t_{90} (min)	V_c (×10^{-5} mol/cm^3)
Unfilled SSBR	0.83	5.11	4.28	4.5	9.3	/
SSBR/silica	4.46	18.84	14.38	3.2	34.9	5.281±0.117
SSBR/silica/L2	3.58	16.76	13.18	3.5	34.4	5.744±0.075
SSBR/silica/L4.5	3.85	19.11	15.26	2.8	38.2	5.829±0.013
SSBR/silica/L10	4.45	20.73	16.28	3.6	37.0	5.954±0.032
SSBR/silica/FL2	3.78	16.77	12.99	3.6	34.5	5.675±0.089
SSBR/silica/FL4.5	3.61	18.02	14.41	2.7	36.8	5.767±0.012
SSBR/silica/FL10	4.18	19.78	15.60	4.3	35.0	5.773±0.023

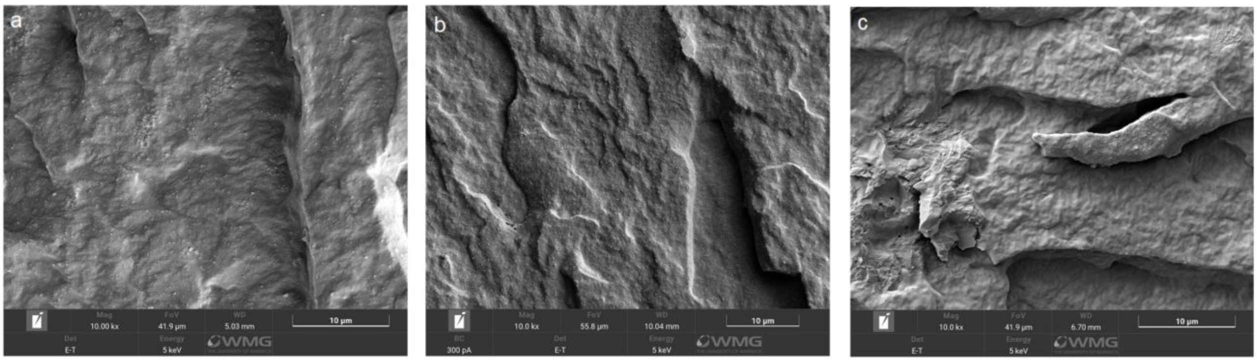

Figure S3 (a) The section morphology of (a) SSBR/silica, (b) SSBR/silica/F1-2 and (c) SSBR/silica/F1-10 vulcanizates

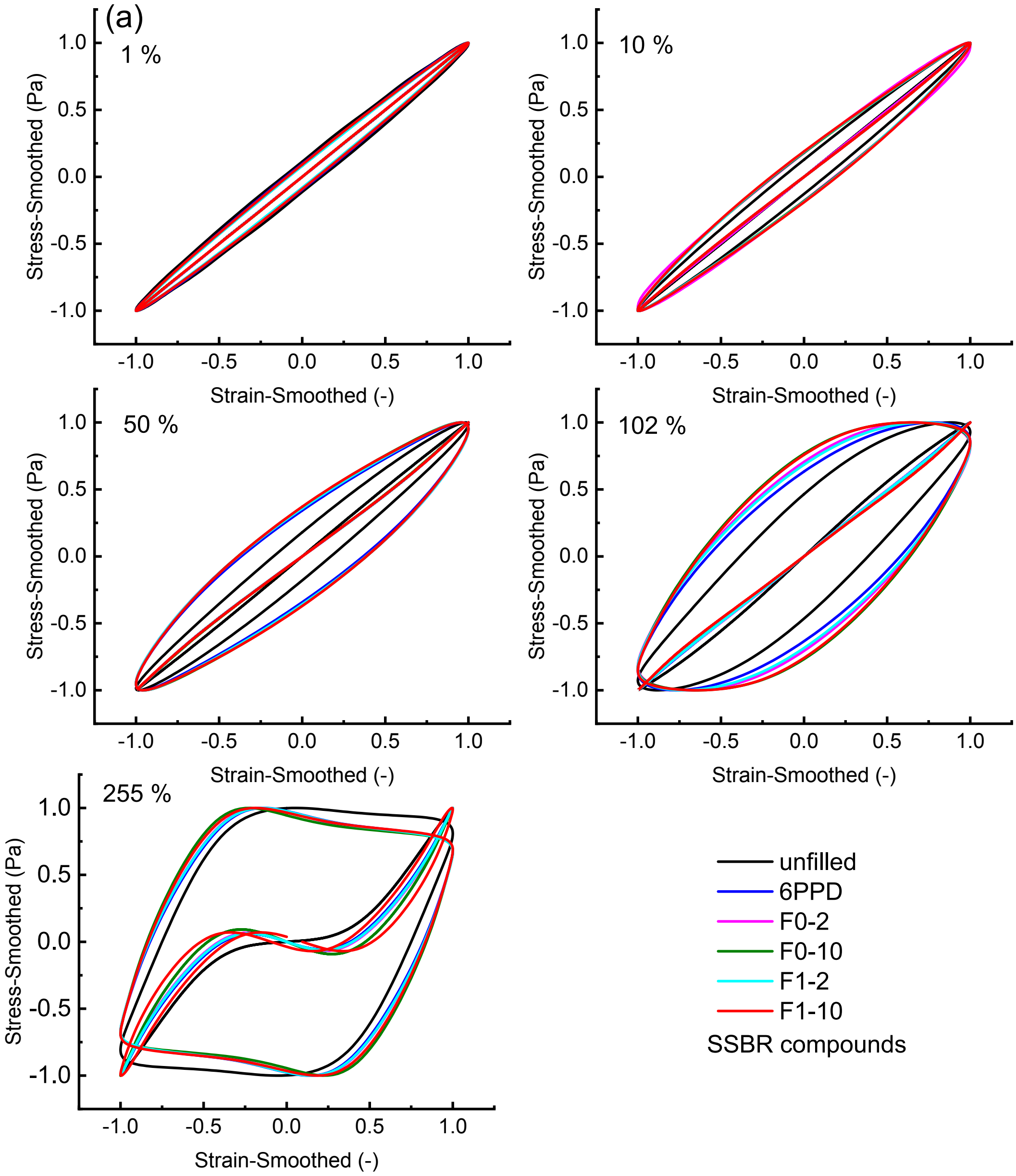

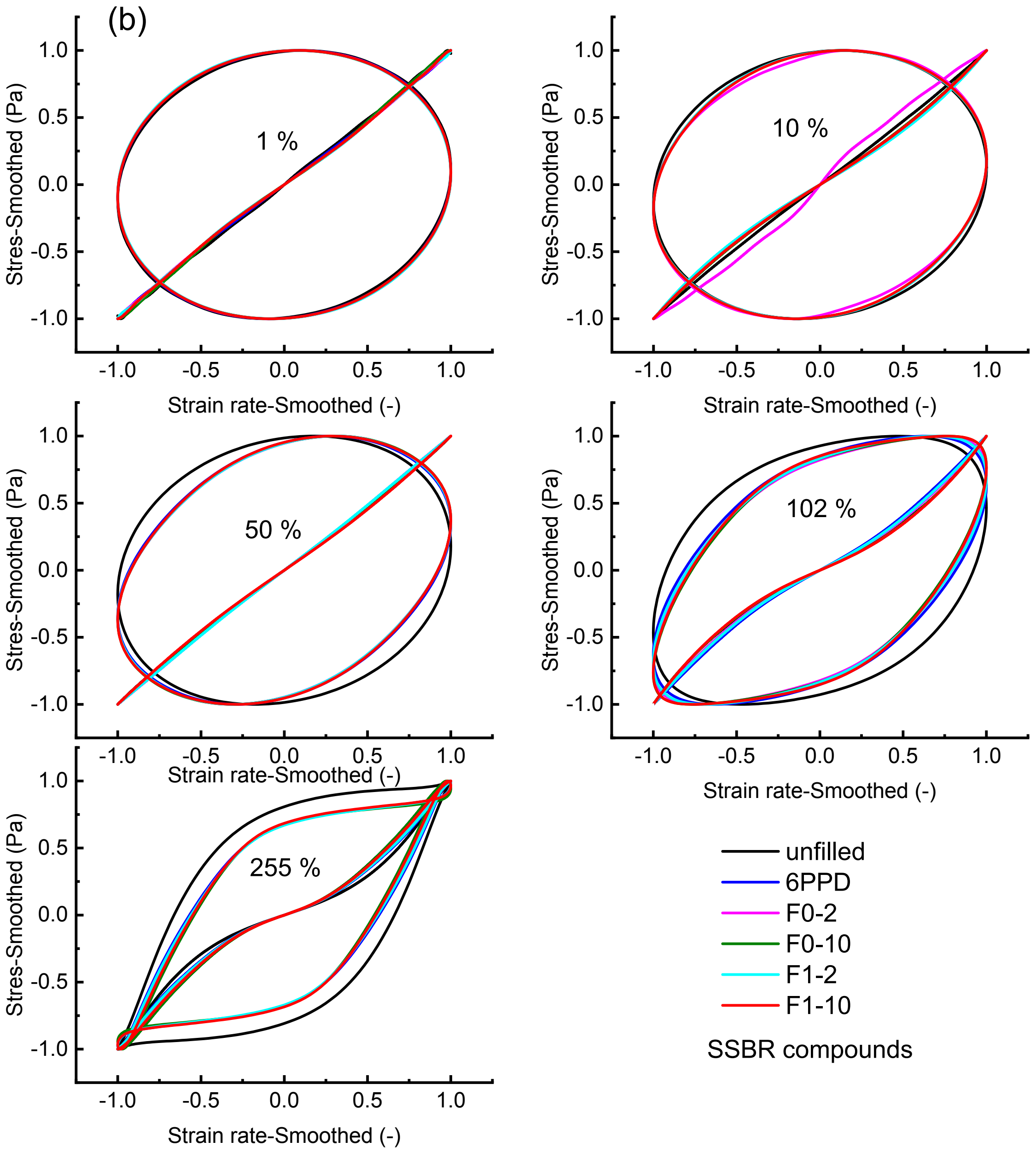

Figure S4 (a) Elastic and (b) Lissajous-Bowditch plots of uncured SSBR compounds based on the strain sweep at 1 Hz and under strains of 1%, 10%, 50%,102% and 255%. Strain and stress data are normalised with the maximum strain/stress in the oscillation cycle. Circles are the plots of the normalised stress signals to the strain, while lines are the normalised elastic stress signals.

Calculation of conversion rate ($d\alpha/dt$)

The $d\alpha/dt$ was calculated by differentiating the degree of conversion (α), which was defined as [1]:

$$\alpha = \frac{S'_t - S'_{min}}{S'_{max} - S'_{min}} \quad (1)$$

where S'_t is the S' value at the time of t. In this study, α value at 60 min was regarded as 1.00, although the vulcanisation did not reach the full level.

Reference

1. Hosseini, S.M. and M. Razzaghi-Kashani, *Vulcanization kinetics of nano-silica filled styrene butadiene rubber.* Polymer, 2014. **55**(24): p. 6426-6434.
